# Comparative transcriptome analyses on terpenoids metabolism in field- and mountain-cultivated ginseng roots

**DOI:** 10.1186/s12870-019-1682-5

**Published:** 2019-02-19

**Authors:** Hang Fan, Ke Li, Fan Yao, Liwei Sun, Yujun Liu

**Affiliations:** 1 National Engineering Laboratory for Tree Breeding, College of Biological Sciences and Biotechnology, Beijing Forestry University, Qinghuadonglu No. 35, Haidian District, Beijing, 100083 China; 2 Research Institute of Advanced Eco-Environmental Protection Technology, Beijing Forestry University, Qinghuadonglu No. 35, Haidian District, Beijing, 100083 China

**Keywords:** Field-cultivated ginseng, Mountain-cultivated ginseng, Transcriptome analysis, Terpenoids biosynthesis genes, Ginsenosides, Terpenoid phytohormones

## Abstract

**Background:**

There exist differences in morphological traits and phytochemical compositions between field- and mountain-cultivated *Panax ginseng* (FCG and MCG), which might be attributed to variations of terpenoids metabolism adapting to different growth conditions. The present work aims to uncover these variations.

**Results:**

Among 26,648 differentially expressed genes, 496 genes distributed in seven dominant terpenoids pathways were identified. Diterpenoids and triterpenoids biosynthesis genes were significantly higher-expressed in FCG root. Conversely, biosynthesis of carotenoids was significantly more active in MCG root. Additionally, terpenoids backbones, monoterpenoids, sesquiterpenoids, and terpenoid-quinones biosyntheses were neither obviously inclined. Our determination also revealed that there were more gibberellins and steroids accumulated in FCG root which might be responsible for its quick vegetative growth, and enriched abscisic acid and germacrenes as well as protopanaxatriol-type ginsenosides might be major causes of enhanced stress-resistance in MCG root.

**Conclusions:**

The study firstly provided an overview of terpenoids metabolism in roots of FCG and MCG in elucidating the underlying mechanisms for their different morphological appearances and phytochemical compositions.

**Electronic supplementary material:**

The online version of this article (10.1186/s12870-019-1682-5) contains supplementary material, which is available to authorized users.

## Background

After being used as an important traditional herb for more than 5000 years, ginseng *(Panax ginseng* Meyer*)* has been widely accepted in Asia [1], and it has been recently adopted also in US Pharmacopeia owing to its miscellaneous health benefits. Ginseng has been proven effective in treating hypodynamia, anorexia, shortness of breath, palpitation, insomnia, impotence, hemorrhage, and diabetes [2, 3]. On one hand, the potent pharmacological properties bring ginseng a considerable and worshipful consumption, but on the other hand, the excessive consumption results in resource of wild ginseng being endangered. To satisfy the ever-increasing consumption, two types of artificially cultivated ginseng, field- and mountain-cultivated ginsengs, which have been recorded since 1600 years ago, are widely developed via modern agricultural technologies.

**Fig. 1:**
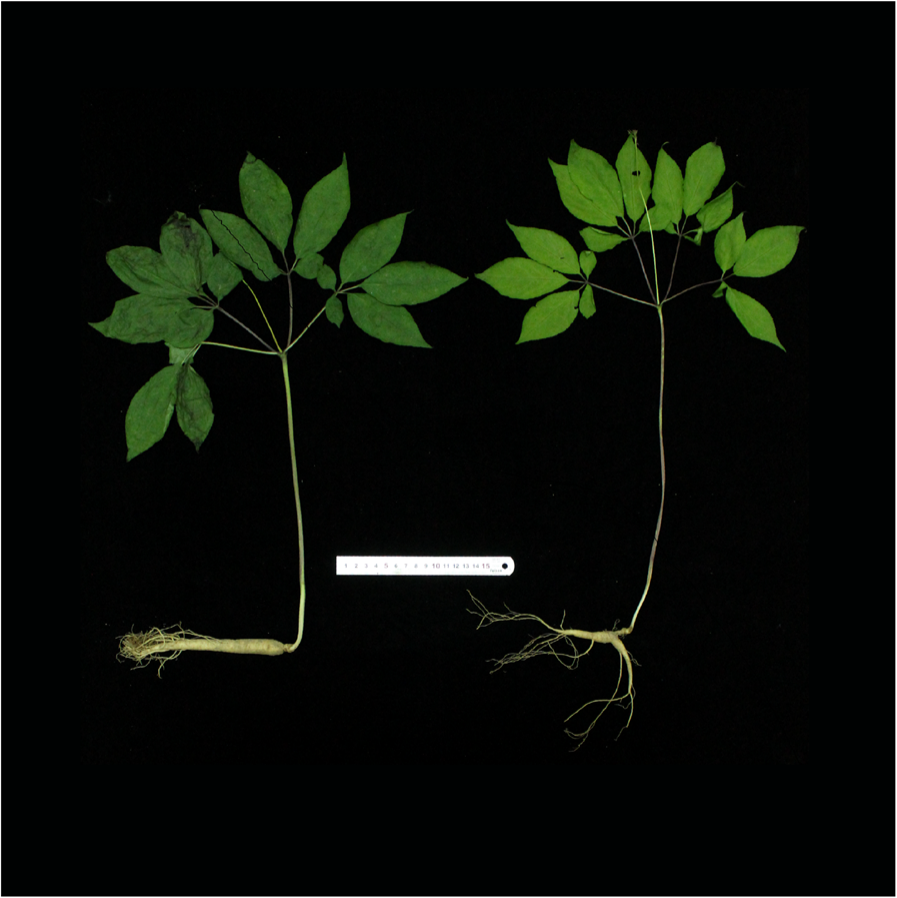
Typical morphologies of field- and mountain-cultivated *Panax ginseng* plants. Left, a 4-year-old field-cultivated ginseng; right, an 8-year-old mountain-cultivated ginseng

Although the two types of ginseng are both sown artificially, they differ in environmental conditions and daily management. Field-cultivated ginseng (FCG) is usually planted in gardens or fields, managed like vegetables, and transplanted to a new fertile bed every two years to sustain its root growth. Conversely, mountain-cultivated ginseng (MCG) is planted imitatively in a natural forest environment without transplantation, scarification, irrigation, or fertilization during the periods of growth, thus suffering much severe environmental stresses as the wild ginseng. Consequently, the differences in growth conditions and daily management contribute to several physiological diversities in morphology and stress-resistance between the two types of ginseng root [4]. Firstly, the main root of FCG is straighter and stronger than that of MCG which possesses more long and branched roots (Fig. 1). It is empirically believed that morphological characteristics of a ginseng root are closely related with its ginsenoside content, and the root with more long branches contained greater amounts of ginsenosides [5], thus, the price of MCG is estimated ~ 10-fold higher than that of FCG. Secondly, normal harvest period of MCG (12–15 years), although shorter than that of the wild ginseng (more than 20 years), is twice longer than that of FCG (5–6 years) which becomes very susceptible to diseases since the seventh year. In other words, MCG exhibits an obvious advantage over FCG on resistances to both biotic (pathogenic and herbivory) and abiotic stresses.

As the most abundant and structurally diverse group of natural products with over 40,000 individual compounds, terpenoids play several important roles in both primary metabolism serving as regulators of plant growth and development (e.g. gibberellins and abscisic acid), electron carriers (e.g. side-chains of ubiquinone and plastoquinone), or elements of membrane structure (e.g. phytosterols) and defense compounds (e.g., volatile compounds) in response to biotic and abiotic stresses [6]. It is widely accepted that terpenoids metabolisms are obviously different between cultivated and wild plants. For instance, Nayyar et al. [7] reported that wild chickpea evolved a potential genetic mechanism of drought tolerance, producing a higher content of abscisic acid, a sesquiterpene, than that of cultivated plants upon water stress. Besser et al. [8] found that trichomes of the cultivated and wild tomatoes showed nearly opposite terpenoid profiles: mainly consisting of monoterpenes and low level of sesquiterpenes in cultivated plants, but very low level of monoterpenes and abundant insecticidal sesquiterpene carboxylic acids in wild plants, which enhances the resistance to pathogens and pests. Taking the topic back to the two types of ginseng, it is speculated that the morphological variation as shown in Fig. 1 might be a reflection of changes in terpenoids metabolism.

Transcriptome analysis has already been deeply applied for investigations on terpenoids metabolism [9–11], especially for discovering variations in terpenoids biosynthesis between cultivated and wild plants. Bleeker et al. [12] compared sesquiterpenoids biosynthesis of a wild (*Solanum habrochaites*) with a cultivated (*S. habrochaites*) tomato species via pyrosequencing, and identified seven and six characteristic sesquiterpene synthase genes, respectively. Tao et al. [13] sequenced a wild (*Gossypium australe*) and a cultivated (*G. arboreum*) cotton species using the Illumina Hiseq 2000 RNA-seq platform, and found that expression of terpenoids synthase family was down-regulated in *G. australe*, resulting in a delayed gland morphogenesis and lower gossypol content in the wild species. With respect to FCG and MCG, a comparative transcriptome analysis are urgently required to be conducted, since little is known about differences in terpenoids metabolism between the two types of ginseng.

Ginsenosides are the most valuable and characteristic triterpenoids responsible for the potent pharmacological properties described above, and their accumulations in FCG and MCG have been widely compared. Yong et al. [4] observed that contents of protopanaxatriol-type ginsenosides including Re, Rf and Rg_1_ were higher in FCG root than in MCG’s that contained higher levels of protopanaxadiol-type ginsenosides such as Rb_1_, Rb_2_, Rc, Rd., and Rh_2_. However, Chen et al. [14] found that contents of the protopanaxadiol-type ginsenosides Rb_1_, Rb_2_, Rc and Rd. and the oleanane-type ginsenoside Ro were higher in FCG root, whereas Rf and a protopanaxadiol-type ginsenoside Ra_3_ enriched in MCG’s. Different from either of the former two researches, Xu et al. [15] presented that both Rf and Rb_2_ were abundant in FCG root, but higher levels of Rb_1_, Rc, Rd. and Ra_3_, the other three protopanaxadiol-type ginsenosides Ra_2_, Ra_7_ and Rs_6_, quinquenoside R_1_, and notoginsenoside Fe were detected in MCG’s, and contents of Re and Rg_1_ showed no significant differences between the two types of ginseng roots. Therefore, to terminate the debate on ginsenosides accumulations in the roots of FCG and MCG, a further comparison based on real-time expression of related genes is expected, considering the biosynthesis of ginsenosides is dynamically changing.

In the current study, we performed a comparative transcriptome analyses of FCG and MCG roots and measured contents of corresponding terpenoids including two phytohormones (gibberellins and abscisic acid), ginsenosides and several other key terpenes. Our analyses attempted to present an overview of terpenoids metabolism in *P. ginseng*, which might help to elucidate the underlying mechanism of the physiological diversities between the roots of FCG and MCG, and also serve as a basis for the future investigation on terpenoids biosynthesis of other species in the *Panax* genus.

## Results

### Transcriptome profiles and functional annotation

#### Transcriptome profiles

To achieve a global overview of gene expression profiles in roots of both FCG and MCG, their cDNA libraries were constructed and sequenced by the Illumina Hiseq4000 platform. After trimming of low-quality reads and blasting with the reference ginseng genome database, 56,233 genes were identified in our transcriptome, including 43,902 genes successfully mapped into reference genome, and 12,331 new genes unmapped but predicted with other three database (6984 genes with NR, 4586 with Swiss-Prot and 1362 with Pfam). Detailed information on quality control and distribution was shown in Additional file 1 Table S3 and Additional file 2: Table S4.

#### GO and COG functional annotations

**Fig. 2:**
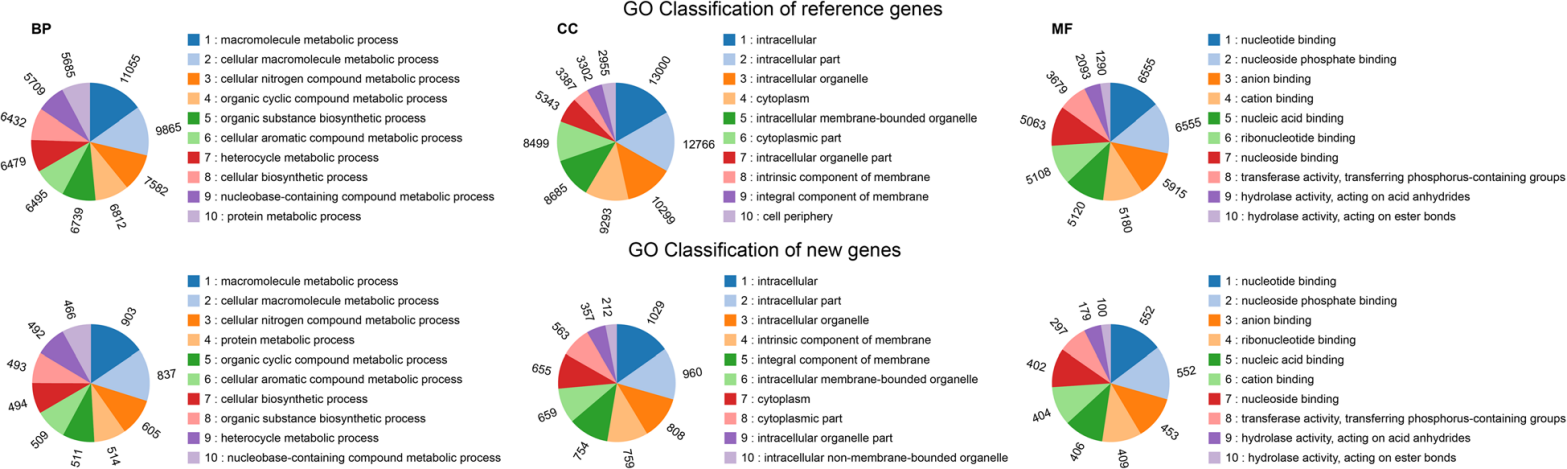
GO classifications of reference and new genes. The results are summarized in three main GO categories: biological process, cellular component, and molecular function. BP, biological process; CC, cellular component; MF, molecular function

GO and COG functional annotations were performed to classify the functions of the 56,233 genes. GO analysis annotated 33,144 genes including 2990 new genes, which could be assigned into 2464 GO terms. The top-10 enriched terms were basically consistent in both GO classifications of reference and new genes (Fig. 2). In the main category of “biological process”, the majority of genes was assigned to terms “macromolecule metabolic process” (That is, 11,958 genes including both reference and new genes were matched in GO classifications; the same below.), “cellular macromolecule metabolic process” (10,702) and “cellular nitrogen compound metabolic process” (8187). With respect to the main category of “cellular component”, the “intracellular” (14,029 genes matched), “intracellular part” (13,726) and “intracellular organelle” (11,107) were the dominant terms. Under the third main category of “molecular function”, the dominant terms were “nucleotide binding” (7107 genes matched), “nucleoside phosphate binding” (7107) and “anion binding” (6368). It is worth mentioning that the terms “secondary metabolic process” and “regulation of secondary metabolic process” annotated 247 and 22 genes, respectively, which might be related to the secondary metabolism concerning biosyntheses of the numerous terpenes in ginseng root.

**Fig. 3:**
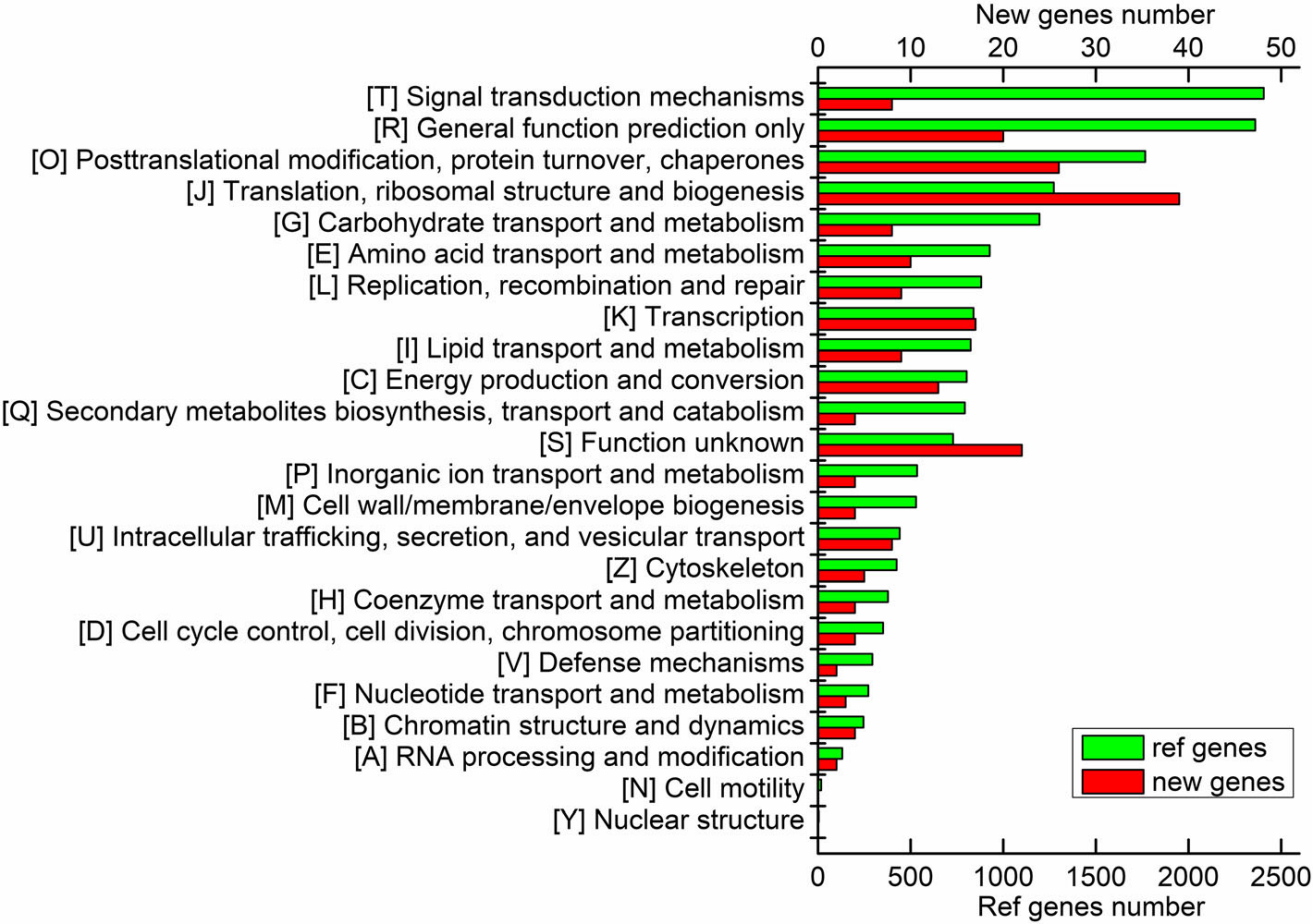
Histogram of COG functional classification. The X axes at the top and bottom present numbers of new and reference genes annotated by COG analysis, respectively. The COG functional classification from top to bottom is sorted from large to small by numbers of reference genes

In addition to GO analysis, functions of the reference and new genes were also annotated by COG analysis based on their putative translated protein sequences. In our COG analysis, 20,953 genes including 672 new genes were assigned to 24 functional classifications (Fig. 3). Among the classifications of reference genes, “signal transduction mechanisms” (2406 genes matched) was the largest group, followed by “general function prediction only” (2360), “post translational modification, protein turnover, chaperone function” (1767), “translation, ribosomal structure and biogenesis” (1273), and “carbohydrate transport and metabolism” (1196). For the new genes, the top five classifications were “translation, ribosomal structure and biogenesis” (39), “post translational modification, protein turnover, chaperone function” (26), “function unknown” (22), “general function prediction only” (20), and “transcription” (17). Again it is noteworthy that a total of 797 reference and new genes was assigned to the classification “secondary metabolites biosynthesis, transport and catabolism”, which might also be associated with the secondary metabolism, especially with that of terpenes, occurred in ginseng roots. Out of the 43,902 genes mapped into the reference genome, 30,154 genes were aligned to GO, 20,281 genes were aligned to COG, and 22,484 genes were aligned to KEGG, and totally 36,577 genes (83.3%) were annotated, which could be adequately representative of the transcriptomes.

### Analysis of DEGs related to terpenoids metabolisms in roots of FCG and MCG

**Fig. 4:**
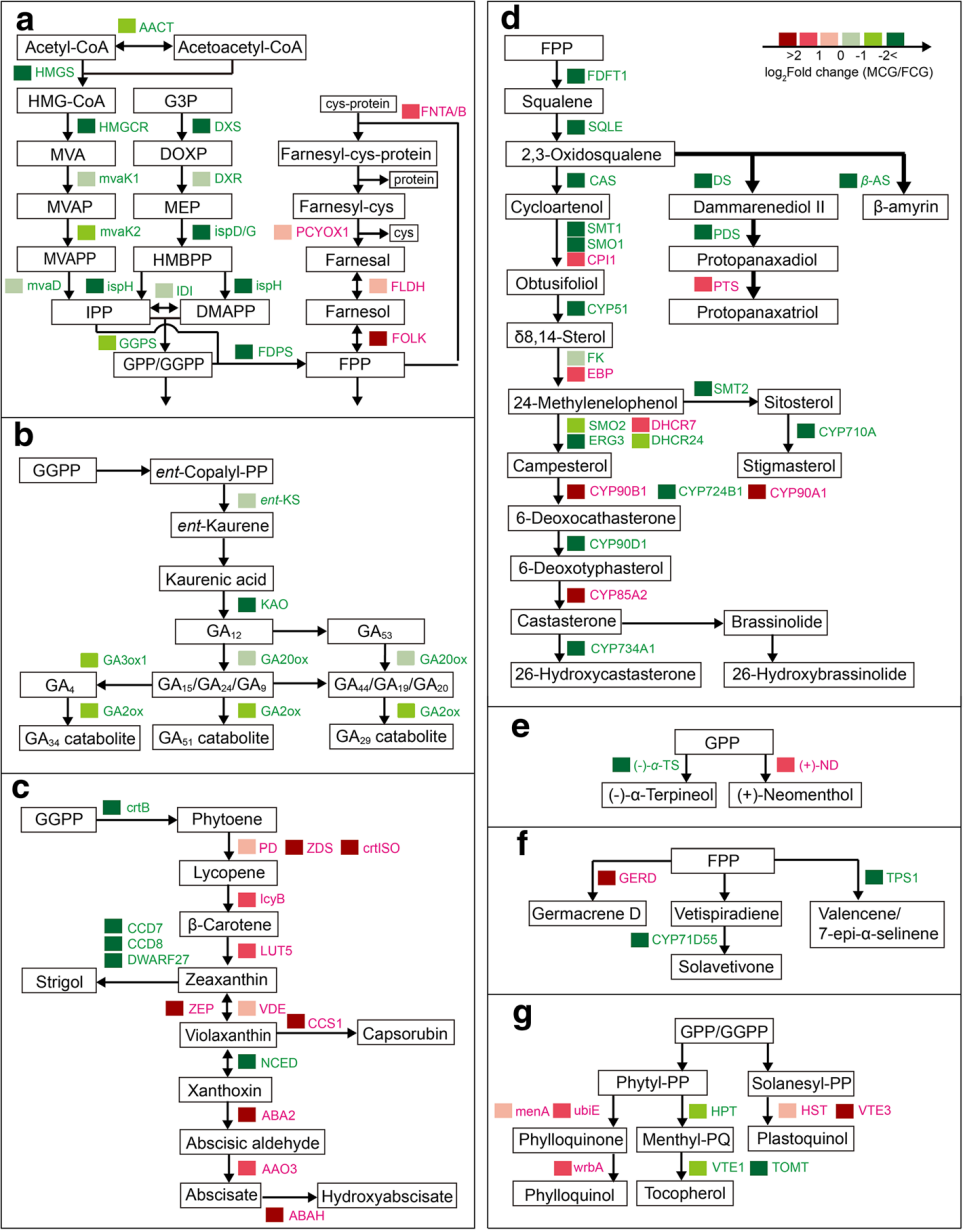
Biosyntheses of various terpenoids in the two types of ginseng root. Acronyms of the enzymes catalyzing corresponding biochemical reactions are written in green and red as indicated by the coloration scale presented to the upright corner of this figure, which were encoded by genes higher-expressed in field- and mountain-cultivated ginseng (FCG and MCG) roots, respectively. **a** Terpenoid backbone biosynthesis: ACT, acetyl-CoA C-acetyltransferase; HMGS, hydroxymethylglutaryl-CoA synthase; HMGCR, hydroxymethylglutaryl-CoA reductase; mvaK1, mevalonate kinase; mvaK2, phosphomevalonate kinase; mvaD, diphosphomevalonate decarboxylase; DXS, 1-deoxy-D-xylulose-5-phosphate synthase; DXR, 1-deoxy-D-xylulose-5-phosphate reductoisomerase; ispD, 2-C-methyl-D-erythritol 4-phosphate cytidylyltransferase; ispG, (*E*)-4-hydroxy-3-methylbut-2-enyl-diphosphate synthase; ispH, 4-hydroxy-3-methylbut-2-en-1-yl diphosphate reductase; IDI, isopentenyl-diphosphate delta-isomerase; GGPS, geranylgeranyl diphosphate synthase; FDPS, farnesyl diphosphate synthase; FNTA/B, alpha/beta-protein farnesyltransferase; PCYOX1, prenylcysteine oxidase; FLDH, NAD^+^ −dependent farnesol dehydrogenase; FOLK, farnesol kinase. **b** Diterpenoids biosynthesis: *ent*-KS, *ent*-kaurene synthase; KAO, ent-kaurenoic acid hydroxylase; GA20ox, gibberellin 20-oxidase; GA3ox1, gibberellin 3-beta-dioxygenase; GA2ox, gibberellin 2-oxidase. **c** Carotenoids biosynthesis: crtB, 15-cis-phytoene/all-trans-phytoene synthase; PD, 15-cis-phytoene desaturase; ZDS, zeta-carotene desaturase; crtISO, prolycopene isomerase; lcyB, lycopene beta-cyclase; LUT5, beta-ring hydroxylase; CCD7, 9-cis-beta-carotene 9′,10′-cleaving dioxygenase; CCD8, carlactone synthase; DWARF27, beta-carotene isomerase; ZEP, zeaxanthin epoxidase; VDE, violaxanthin de-epoxidase; CCS1, capsanthin/capsorubin synthase; NCED, 9-cis-epoxycarotenoid dioxygenase; ABA2, xanthoxin dehydrogenase; AAO3, abscisic-aldehyde oxidase; ABAH, (+)-abscisic acid 8′-hydroxylase. **d** Triterpenoids biosynthesis: FDFT1, farnesyl-diphosphate farnesyltransferase; SQLE, squalene monooxygenase; CAS, cycloartenol synthase; DS, dammarenediol-II synthase; *β*-AS, beta-amyrin synthase; PDS, protopanaxadiol synthase; PTS, protopanaxatriol synthase; SMT1, sterol 24-C-methyltransferase; SMO1, 4,4-dimethyl-9beta,19-cyclopropylsterol-4alpha-methyl oxidase; CPI1, cycloeucalenol cycloisomerase; CYP51, sterol 14-demethylase; FK, delta14-sterol reductase; EBP, cholestenol Delta-isomerase; SMO2, 4-alpha-methyl-delta7-sterol-4alpha-methyl oxidase; ERG3, delta 7-sterol-5-desaturase; SMT2, 24-methylenesterol C-methyltransferase; DHCR7, 7-dehydrocholesterol reductase; DHCR24, delta24-sterol reductase; CYP710A, steroid 22-desaturase; CYP90B1, steroid 22-alpha-hydroxylase; CYP724B1, cytochrome P450 family 724 subfamily B polypeptide 1; CYP90A1, cytochrome P450 family 90 subfamily A polypeptide 1; CYP90D1, cytochrome P450 family 90 subfamily D1; CYP85A2, cytochrome P450 family 85 subfamily A2; CYP734A1, PHYB activation tagged suppressor 1. **e** Monoterpenoids biosynthesis: (−)-*α*-TS, (−)-alpha-terpineol synthase; (+)-ND, (+)-neomenthol dehydrogenase; menA, 1,4-dihydroxy-2-naphthoate octaprenyltransferase. **f** Sesquiterpenoids biosynthesis: GERD, (−)-germacrene D synthase; CYP71D55, premnaspirodiene oxygenase; TPS1, valencene/7-epi-alpha-selinene synthase. **g** terpenoid-quinone biosynthesis: menA, 1,4-dihydroxy-2-naphthoate octaprenyltransferase; ubiE, demethylmenaquinone methyltransferase; wrbA, NAD(P)H dehydrogenase; HPT, homogentisate phytyltransferase; VTE1, tocopherol cyclase; TOMT, tocopherol O-methyltransferase; HST, homogentisate solanesyltransferase; VTE3, 2-methyl-6-phytyl-1,4-hydroquinone methyltransferase

**Fig. 5:**
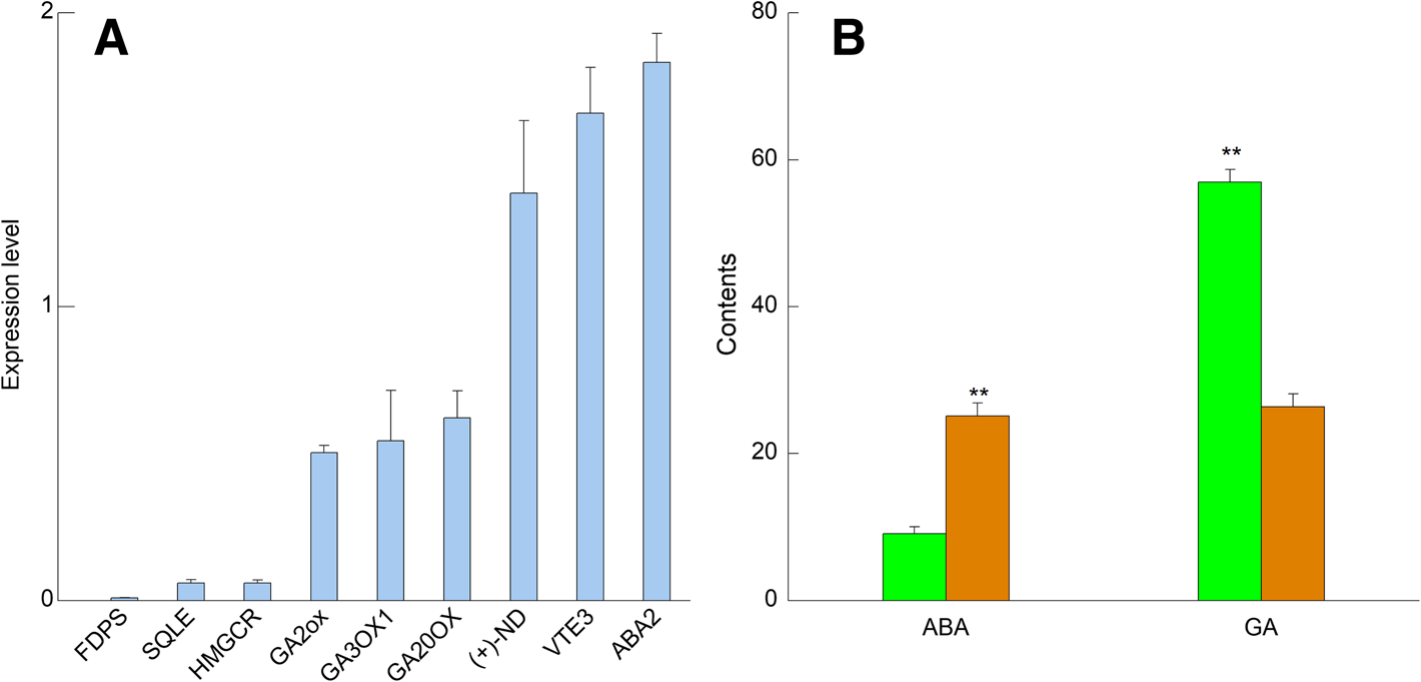
Validations of transcriptome analyses of the two types of ginseng root. **a** Expression levels of nine randomly selected differentially expressed genes (DEGs) determined by qRT-PCR. Gene expressions calculated by fold change of 2 ^– (ΔCtMCG-ΔCtFCG)^ below (the left side six columns) and above (the right side three columns) 1.0 indicated those genes being significantly higher-expressed in field- and mountain-cultivated ginseng (FCG and MCG) roots, respectively. FDPS, farnesyl diphosphate synthase; HMGCR, hydroxymethylglutaryl-CoA reductase; SQLE, squalene monooxygenase; GA20ox, gibberellin 20-oxidase; GA3ox1, gibberellin 3-beta-dioxygenase; GA2ox, gibberellin 2-oxidase; VTE3, MPBQ/MSBQ methyltransferase; ABA2, xanthoxin dehydrogenase; (+)-ND, (+)-neomenthol dehydrogenase. **b** Contents of ABA and GA in the two types of ginseng root. Columns in green and yellow represent FCG and MCG roots, respectively. Contents of ABA and GA are expressed as ng/g f.w. (*n* = 3). “**” indicates extremely significant difference (*P* < 0.01)

Among the 56,233 genes, there were 15,509 and 11,139 genes significantly higher-expressed in FCG and MCG roots, respectively, which were regarded as DEGs (|log_2_FC (FPKM _MCG_/FPKM _FCG_)| > 1 and *P* value < 0.05). To disclose the difference in terpenoids metabolisms between FCG and MCG roots, we performed KEGG analysis and found that 496 DEGs were involved in terpenoids-related pathways. Thereinto terpenoids structural units biosynthesis and all directly associated downstream pathways were the main emphasis of our investigation, such as “terpenoid backbone biosynthesis (ko00900)”, “monoterpenoid biosynthesis (ko00902)”, “diterpenoid biosynthesis (ko00904)”, “carotenoid biosynthesis (ko00906)”, “sesquiterpenoid and triterpenoid biosynthesis (ko00909)”, “steroid biosynthesis (ko00100)”, and “ubiquinone and other terpenoid-quinone biosynthesis (ko00130)” (Fig. 4). Validation of corresponding key genes in these terpenoids biosynthesis pathways was performed through qRT-PCR, of which expressions were in line with our transcriptional analysis (Fig. 5a).

#### Biosyntheses of the terpenoid backbone

As shown in Fig. 4a, it was found that terpenoids backbone biosynthesis in ginseng root involved 58 DEGs that encoded 18 enzymes with those written in green being significantly higher-expressed in FCG root and those in red significantly higher-expressed in MCG root. Usually, biosynthesis of a terpenoid backbone resulted in a universal building block, isopentenyl pyrophosphate (IPP), that is generated from two discrete biosynthetic pathways: the mevalonic acid (MVA) pathway in cytosol and the 2-*C*-methyl-D-erythritol 4-phosphate/1-deoxy-D-xylulose 5-phosphae (MEP/DOXP) pathway in plastids [16]. With the MVA pathway (at the left of Fig. 4a), IPP was biosynthesized from acetyl-CoA by sequential actions of the enzymes AACT, HMGS, HMGCR, mvaK1, mvaK2 and mvaD. In case of the MEP/DOXP pathway (at the middle of Fig. 4a), IPP could be formed from glyceraldehyde 3-phosphate (G3P) via a series of reactions catalyzed by the enzymes DXS, DXR, and ispD/G/H. Thereafter IPP or its isomer dimethylallyl pyrophosphate (DMAPP) was further transformed into geranyl diphosphate (GPP), geranylgeranyl diphosphate (GGPP) and farnesyl diphosphate (FPP) by the enzymes GGPS and FDPS that are origins of all terpenoids biosyntheses pathways. According to their FPKM values, genes related to these two IPP biosynthesis pathways and consequently their two downstream enzymes, GGPS and FDPS, were significantly higher-expressed in FCG root than in MCG’s, suggesting that biosyntheses of terpenoid backbones through these two pathways might be more active in FCG than in MCG.

Except for directly synthesizing downstream terpenoids, as we saw at the right of Fig. 4a, some FPP participated in protein isoprenylation that occurs on cell membranes (Bhandari et al., 2010). In details, a FPP conjugated to a cysteine of a protein via C-S bonds and produced a farnesyl-cys-protein complex by the enzymes FNTA/B. Then the molecular farnesyl-cys was separated from *C*-terminal of the protein and oxidized to farnesal by the enzyme PCYOX1, which was subsequently dehydrogenized into farnesol by the NAD^+^-dependent enzyme FLDH and ultimately phosphorylated into FPP by the enzyme FOLK. It was worth noting that genes encoding such four key enzymes as FNTA, PCYOX1, FLDH and FOLK (at the right of Fig. 4a; written in red) showed significantly higher expressions in MCG root than in FCG’s, suggesting that the protein isoprenylation was more active in MCG than in FCG.

#### Diterpenoids biosynthesis: responsible for the rapid growth of FCG under optimal conditions

Totally five enzymes encoded by 21 DEGs were found in diterpenoid biosynthesis (Fig. 4b). Firstly, through catalization of the enzyme *ent*-KS, *ent*-copalyl-PP derived from GGPP (Fig. 4a) was transformed to kaurenic acid which generated GA_12_ by the enzyme KAO. Subsequently, GA_12_ was catalyzed by the enzyme GA20ox, producing a series of GA isoforms including GA_15_, GA_24_, GA_9_, GA_53_, GA_44_, GA_19_ and GA_20_. Among which, GA_9_ is transformed into GA_4_ by the enzyme GA3ox1, and GA_4_, GA_9_ and GA_20_ are all catalyzed by the enzyme GA2ox to form GA_34_-, GA_51_- and GA_29_-catabolites, respectively. Overall, these five enzymes (*ent*-KS, KAO, GA20ox, GA3ox1 and GA2ox) were significantly higher-expressed in FCG root, implying that biosynthesis of diterpenoids, especially GAs, was more active in FCG root. As a further proof, it was measured that content of GAs in FCG root (56.962 ng/g f.w.) was about twice of that in MCG root (26.351) (Fig. 5b), which might be responsible for the rapid growth of FCG root under optimal field conditions.

#### Carotenoids biosynthesis: Responsible for coping with harsh conditions faced by MCG

As Fig. 4c shows, 34 DEGs encoding 16 enzymes were found in the carotenoids biosynthesis pathway. Firstly, five enzymes, namely, crtB, PD, ZDS, crtISO and lcyB, which jointly converted GGPP (Fig. 4a) to β-carotene, were mainly higher-expressed in MCG root than in FCG’s. Secondly, β-carotene was inclined to form zeaxanthin in MCG root by the enzyme LUT5 but strigol in FCG root via three steps through the three enzymes DWARF27, CCD7 and CCD8 whose higher expressions in FCG root might indicate a more active biosynthesis of the newly discovered phytohormone strigolactone. Thirdly, zeaxanthin was transformed to capsorubin by the enzyme CCS1 or violaxanthin by the enzymes ZEP and VDE. Hereinto, expressions of ZEP and VDE were notably higher in FCG root than in MCG’s, however, a higher expression of CCS1 in MCG root was detected. Finally, violaxanthin was transformed into xanthoxin through the enzyme NCED, which was catalyzed into abscisate through the enzymes ABA2 and AOA3, and further hydroxylated to 8′-hydroxyabscisic acid through the enzyme ABAH. And these three enzymes were also higher-expressed in MCG root than in FCG’s. These results suggest that there might be a higher accumulation of ABA to cope with the harsh conditions faced by the MCG root. To verify this hypothesis, ABA in roots of FCG and MCG were determined (Fig. 5b), and it was found that the content of ABA in MCG root (25.100 ng/g f. w.) was 2.6 times higher than that in FCG’s (9.510 ng/g f.w.), being consistent with our deduction inferred from the above transcriptome analysis.

An interesting finding should be mentioned on the correlation between the MVA/MEP pathways and its downstream ABA biosynthesis. It is generally recognized that both GA and ABA biosyntheses relies on the FPP originated from the MVA/MEP pathways [17]. However, this notion was not totally validated by the current work. Even though the GAs biosynthesis (Fig. 4b; note those enzymes in green) indeed showed a similar trend with MVA/MEP pathways (left and middle of Fig. 4a; note those enzymes in green) as predicted, proving that GAs biosynthesis was positively correlated with MVA/MEP pathways, the ABA biosynthesis (Fig. 4c; note those enzymes in red) displayed a conversed trend with MVA/MEP pathways but a same trend with the protein isoprenylation (at the right of Fig. 4a; note those enzymes in red) which were reported to negatively regulated MVA/MEP pathways [17, 18], suggesting that ABA biosynthesis might correlated with the protein isoprenylation pathway more than the MVA/MEP pathways. Namely, it is deduced that a more vigorous biosynthesis of ABA in MCG root might be originated from the FPP that have been circulated via the protein isoprenylation pathway, which obviously needed to be proven in further research.

#### Triterpenoids biosynthesis: Covering biosyntheses of steroids and ginsenosides in ginseng roots

As shown in Fig. 4d, squalene derived from FPP, was oxidized to 2,3-oxidosqualene by the enzyme SQLE, which further converted to steroids and ginsenosides. For steroids biosynthesis, 2,3-oxidosqualene was mainly converted to cycloartenol which was subsequently transformed into stigmasterol and campesterol that participated in biosynthesis of brassinolide (BR). In detail, campesterol was converted to castasterone by a series of enzymes including CYP90B1, CYP724B1, CYP90A1 and CYP90D1, and eventually to brassinolide by the enzyme CYP85A2. As marked in Fig. 4d, most DEGs encoding enzymes before campesterol were significantly higher-expressed in FCG root than in MCG’s, with only the enzymes CPI1, EBP and DHCR7 as exceptions; however, an ambiguous trend was observed among enzymes from CYP90B1 to CYP734A1.

In case of biosynthesis of ginsenosides, the DEGs encoding two saponin synthases, β-AS and DS, which produced β-amyrin and dammarenediol II in respective, were significantly higher-expressed in FCG root than in MCG’s, indicating that β-amyrin and dammarenediol II were more abundant in FCG root. Dammarenediol II was catalyzed by the oxidoreductases PDS and PTS into protopanaxadiol and protopanaxatriol in successive, which were further transformed into ginsenosides (PPD- and PPT-type). In our analyses, PDS gene was significantly higher-expressed in FCG root, but PTS gene showed a converse trend, revealing that biosynthetic activity of PPD-type ginsenosides might be higher in FCG root, meanwhile that of PPT-type ginsenosides were ambiguous, probably at a same level in the two types of ginseng root, or also at a higher level in MCG root than in FCG’s, due to the high level of PPD-type ginsenosides as the substrate. To verify this hypothesis, contents of ginsenosides were determined (Table 1). It was found that PPD-type ginsenosides in MCG root (134.558 mg/g f.w.) were extremely significantly lower than that in FCG’s (189.081), whereas the PPT-type ginsenosides exhibited no significant difference between MCG (162.596) and FCG (160.941) roots. Accordingly, total ginsenosides in MCG root (297.15) was extremely significantly lower than that in FCG’s (350.02). These results were also in line with those of determinations of genes expression (Fig. 4d).

**Table 1:** Contents of ginsenosides in roots of field- and mountain-cultivated ginsengs

Ginsenosides		MCG	FCG
PPD-type ginsenosides	Rc	31.600 ± 1.242	34.553 ± 1.098*
	Rb_2_	26.693 ± 1.407	39.323 ± 0.673**
	Rb_3_	8.060 ± 0.465	10.606 ± 0.159**
	Rb_1_	37.093 ± 4.422	64.100 ± 5.982**
	Rd	17.925 ± 2.887	21.427 ± 0.790
	Rh_2_	13.188 ± 0.106	19.071 ± 0.613**
	total	134.558 ± 10.530	189.081 ± 9.315**
PPT-type ginsenosides	Rg_1_	72.615 ± 1.711**	55.210 ± 0.872
	Re	49.430 ± 3.647	60.201 ± 0.790**
	Rf	21.462 ± 0.332	26.092 ± 1.375**
	Rg_2_	12.340 ± 1.348	13.588 ± 1.611
	F_1_	6.750 ± 1.518	5.849 ± 0.652
	total	162.596 ± 8.556	160.941 ± 5.300

Contents of ginsenosides are expressed as mg/g f.w. (*n* = 3). PPD-type ginsenosides, protopanaxadiol-type ginsenosides; PPT-type ginsenosides, protopanaxatriol-type ginsenosides. “*” and “**” indicated a significant (*P* < 0.05 and > 0.01) and an extremely significant (*P* < 0.05 and > 0.01) differences, respectively

#### Several types of other terpenoids: Relevant to volatile compounds in ginseng

Except forming phytohormones and ginsenosides, the terpenoids backbones were also related to biosyntheses of several types of other terpenoids including “monoterpenoids”, “sesquiterpenoids” and “terpenoid-quinones” (Figs. 4e-g). Monoterpenoids were derived from GPP (Fig. 4a) and converted into (−)-α-terpineol and (+)-neomenthol, whose synthases [Fig. 4e; (−)-α-TS and (+)-ND] were highly expressed in FCG and MCG roots, respectively. In biosynthesis of sesquiterpenoids, FPP (Fig. 4a) directly generated sesquiterpenoids including germacrene, solavetivone and valencene/7-epi-α-selinene in our detection, among which enzymes encoding valencene/7-epi-α-selinene and solavetivone (TPS1 and CYP71D55 in respective) were significantly higher-expressed in FCG root while the enzyme encoding germacrene D (GERD) were significantly higher-expressed in MCG root (Fig. 4f). As to biosyntheses of terpenoid-quinones, GPP (Fig. 4a) was transformed into phytyl-PP and solanesyl-PP, with the former being further converted to phylloquinol or tocopherol and the latter being catalyzed into plastoquinol. The biosyntheses of phylloquinol and plastoquinol were more active in MCG root, with the synthases menA, ubiE, wrbA, HST and VTE3 being higher-expressed, and conversely the synthases of tocopherol, such as HPT, VTE1 and TOMT, were significantly higher-expressed in FCG root (Fig. 4g).

To support our conclusion, GC-MS was performed to profile these terpenoids in the two types of ginseng roots. As shown in Table 2, totally 33 compounds were identified, and their detailed peaks information and total ion chromatography were shown in Additional file 3: Table S5 and Additional file 4: Figure S2. In summary, 10 sesquiterpenoids and 15 triterpenoids accounted for a majority of these compounds, which displayed a dominant accumulation in MCG and FCG roots, respectively. Among which germacrene D was inclined to accumulate in MCG root; alpha-amyrin, gamma-sitosterol, cycloartenol acetate, ergosterol (that is relevant to obtusifoliol and 8,14-sterol), cholesterol chloroformate (relevant to 24-methylenecholesterol), 4-campestene-3-one (relevant to campesterol), and stigmasterol derivatives (including stigmasta-3, 5-dien-7-one, stigmasta-3,5-diene, stigmasta-5, 22-dien-3-ol, stigmasta-5, 22-dien-3-ol, acetate and stigmast-5-en-3-ol, acetate) were highly detected in FCG root. In addition, *p*-menthane that is the upstream product of (+)-neomenthol in monoterpenoids biosynthesis, and 1,4-naphthoquinone that is the upstream product of phylloquinol in terpenoid-quinone biosynthesis, both showed a higher level in MCG root. And conversely, the content of tocopherol was higher in FCG root. These result was also in according with the above transcriptome analysis.

**Table 2:** Comparison of key terpenoids detected by GC-MS

	CAS	Compounds	Name	Ratio of content^a^
Monoterpenoids	61142–32-3	C_10_H_18_	1,3-dimethyl-2-(1-methylethyl)-cyclopentene	F^b^
	99-82-1	C_10_H_20_	p-menthane	1.279
Sesquiterpenoids	25246–27-9	C_15_H_24_	alloaromadendrene	M^b^
	515-13-9	C_15_H_24_	beta-elemene	M
	17334–55-3	C_15_H_24_	calarene	2.454
	56633–28-4	C_15_H_24_	alpha-panasinsene	M
	56684–96-9	C_15_H_24_	beta-neoclovene	M
	23986–74-5	C_15_H_24_	germacrene D	M
	489–39-4	C_15_H_24_	aromandendrene	M
	21747–46-6	C_15_H_24_	ledene	M
	29873–99-2	C_15_H_24_	gamma-elemene	1.688
	23445–02-5	C_15_H_26_O	cubebol	M
Diterpenoids	1686-63-1	C_20_H_30_O	isopimaral	0.473
	1686-54-0	C_21_H_32_O_2_	1-phenanthrenecarboxylic acid	M
Triterpenoids	57–87-4	C_28_H_44_O	ergosterol	0.625
	7144-08-3	C_28_H_45_ClO_2_	cholesterol chloroformate	0.903
	67–96-9	C_28_H_46_O	dihydrotachysterol	0.608
	51014–22-3	C_28_H_46_O	4-campestene-3-one	0.516
	2034-72-2	C_29_H_46_O	stigmasta-3,5-dien-7-one	0.236
	79897–80-6	C_29_H_48_	stigmasta-3,5-diene	M
	83–48-7	C_29_H_48_O	stigmasta-5,22-dien-3-ol	0.682
	83–47-6	C_29_H_50_O	gamma-sitosterol	0.677
	638–95-9	C_30_H_50_O	alpha-amyrin	0.313
	473–98-3	C_30_H_50_O_2_	lup-20 (29)-ene-3,28-diol	0.726
	4651-48-3	C_31_H_50_O_2_	stigmasta-5,22-dien-3-ol, acetate	0.889
	1449-09-8	C_31_H_52_O	24-methylenecycloartano	0.396
	915–05-9	C_31_H_52_O_2_	stigmast-5-en-3-ol, acetate	0.931
	1617-68-1	C_32_H_52_O_2_	lupeol acetate	F
	1259-10-5	C_32_H_52_O_2_	cycloartenol acetate	0.85
Terpenoid-quinone	59–02-9	C_29_H_50_O_2_	tocopherol	0.514
	29790–47-4	C_50_H_70_O_2_	1,4-naphthoquinone	1.536
Others	25061–77-2	C_14_H_12_O_2_	9,10-phenanthrenediol	F
	72800–72-7	C_17_H_24_O_2_	panaxydol	0.483

^a^Ratio of content was calculated based on ion peaks area of MCG root to that of FCG root. The ratio numbers colored in green and red represented the compound was higher-accumulated in FCG and MCG roots, respectively

^b^Letters ‘F’ and ‘M’ indicate those compounds detected only in FCG and MCG, respectively

### TFs annotation

**Fig. 6:**
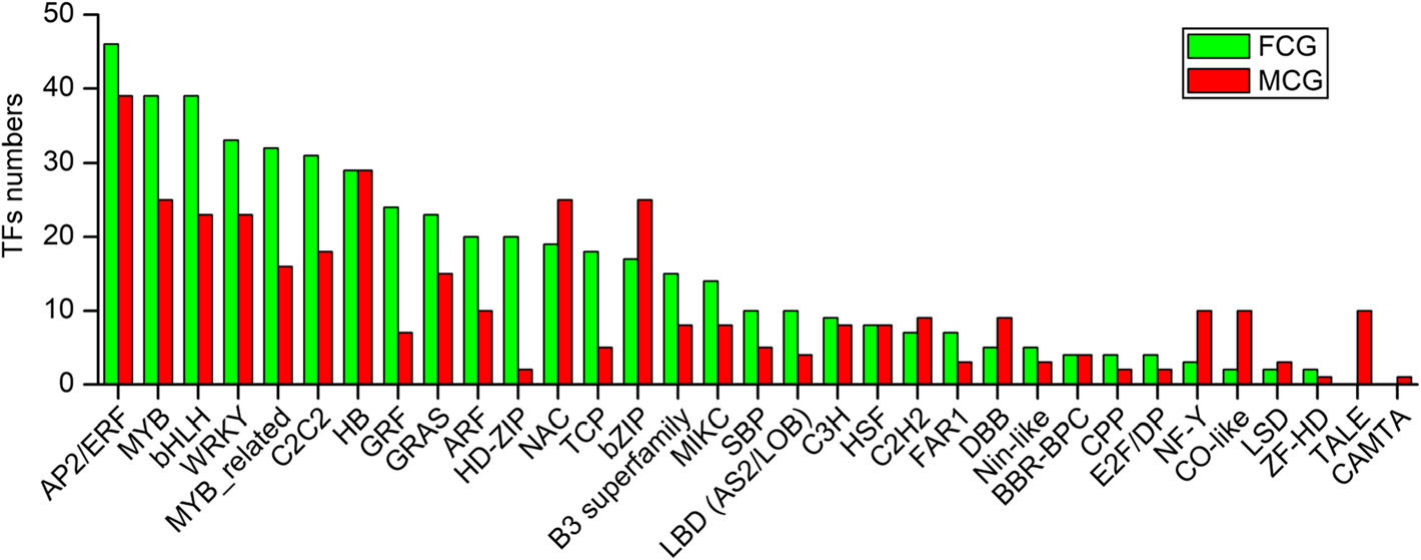
Distribution of differentially expressed TFs in 33 TF families. Y axis represented the numbers of TFs in each family. The columns in green and red meant the TFs were significantly higher-expressed in FCG and MCG, respectively. At the X axis, families of the TFs from left to right are sorted from large to small by numbers of them higher-expressed in FCG root

As a major part of gene expression regulatory components, a total of 3134 TFs relating genes within 44 TF families were annotated, among which the most frequently occurring was AP2/ERF, followed by MYB, bHLH and NAC (Additional file 5: Table S6). With respect to differentially expressed TFs (Fig. 6), 572 and 370 TFs from 33 TF families were significantly higher-expressed in FCG and MCG roots, respectively. Among which, 285 and 233 TFs, more than halves, were classified into AP2/ERF, HB, MYB, NAC, bZIP, bHLH, WRKY and C2C2 families that might regulate the different gene expression between FCG and MCG. Additionally, all TFs in the two families TALE and CAMTA were up-regulated in MCG root, suggesting the two families might associate with stress resistance that were faced by the MCG plants.

## Discussion

**Fig. 7:**
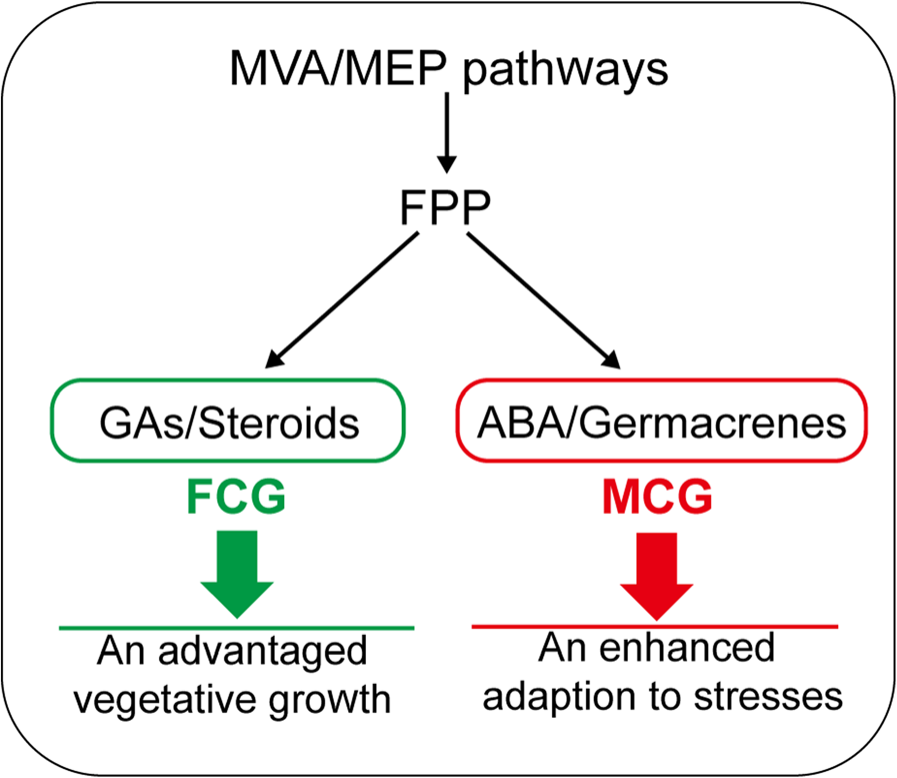
A conceptual model for difference in terpenoids biosyntheses between FCG and MCG. The terpenoids in green and red box indicate an active biosynthesis in FCG and MCG, respectively

Since Kim et al. [19] reported the first transcriptome from the leaf of ginseng, comparative transcriptome analyses have also been widely conducted on different ginseng roots, such as roots of two Korean ginseng cultivars (i.e., cultivars *Cheonryang* and *Yunpoong*) and American ginseng (*P. quinquefolius*, Voucher no. MPS002310) [20], adventitious roots from five Korean ginseng cultivars (i.e., cultivars *Chunpoong*, *Cheongsun*, *Sunhyang*, *Goopong*, and *Sunun*) [21], or four parts of the root of the Korean ginseng cultivar *Chunpoong* (i.e., whole root, main root body, rhizome, and lateral root) [22], providing new insights into the primary and secondary metabolisms of ginseng root. Specific to comparative transcriptome analyses of FCG and MCG roots, several key genes in terpenoids biosynthesis have been reported. Sathiyamoorthy et al. [23] firstly reported comparative transcriptome analyses of FCG and MCG, and preliminarily discussed putative isoprenoids pathways based on 6757 expressed sequence tags from hairy roots and roots of 14-year-old MCG and 4-year-old FCG. Zhen et al. [24] built a set of root transcriptomes from three types of ginseng roots (i.e., roots of FCG, MCG and wild ginseng), and found that three genes encoding key terpenoids biosynthesis enzymes including HMGCR, MVK and SE were more active in wild ginseng root than in FCG’s. Wang et al. [25] identified 78 genes of 13 enzymes from roots of 5-, 12-, 18- and 25-year-old MCG and 4-year-old FCG involved in biosyntheses of terpenoids backbone and triterpenoids. In the present work, among 26,648 DEGs, 496 genes distributed in seven dominant terpenoids pathways. Diterpenoids and triterpenoids biosyntheses genes were significantly higher-expressed in FCG root than in MCG’s. Conversely, biosynthesis for carotenoids were significantly more active in MCG root than in FCG’s. Additionally, terpenoids backbones, monoterpenoids, sesquiterpenoids and terpenoid-quinones biosyntheses were neither obviously inclined. Combined with quantitative determination, we finally deduced an obviously different inclinations of several terpenoids in FCG and MCG roots, depending on the growth conditions and resulting in a diversity of physiological phenotypic traits (Fig. 1). For MCG under harsh conditions, ABA and germacrene D as well as its geometric isomers were notably higher-accumulated. Among which, ABA is well known responsible for mediating responses to drought and salinity stress in roots [26]. Germacrene D and its geometric isomers, which was also detected in ginseng root previously [27] and was proven to be a remarkable enrichment under chilling stress [28], were also conductive to adapt to diverse stresses. Moreover, the adaption to stress was even reflected by the distribution of certain TFs. For instance, the TFs families of NAC, bZIP, NF-Y and CO-like, which were widely recognized to be related to stress resistance [29, 30], were found to be enriched in MCG (Fig. 6). In particular, expressions of the TALE and CAMTA families were only observed in MCG. It had been reported that TALE families, which regulates root growth [31], were up-regulated under low or high temperature stress in rubber and soybean, respectively [32, 33]. Likewise, TFs in the CAMTA family were proven to play a role on roots of soybean and strawberry in response to abiotic stress [34, 35]. Therefore, it could be concluded that these active transcription factors families were associated with enhanced stress resistance in MCG. In FCG under optimal conditions of artificial cultivation, more amount of FPP originated from MVA/MEP pathways might be used for the biosyntheses of GAs and steroids as illustrated in Fig. 7. Since GAs have been widely recognized in promoting root growth [36], higher levels of GAs in FCG than that in MCG could be expected to be enlisted for thickening growth of the main root. In addition, steroids-like campesterol, which stimulates growth of aerial part of the plant [37], and stigmasterol, which affects the expression of genes involved in cell expansion and cell division [38], were enriched in FCG (Table 2). These might be all contributing to an advantaged vegetative growth of its main root with higher biomass thus higher commercial value. To prove this hypothesis, morphologies of FCG and MCG were observed. By comparing their overground and underground portions, as predicted, it is indeed obvious that FCG was significantly superior to MCG on both stems, compound leaves, flower stalks, and petioles, but was obviously less in branches of lateral roots (Fig. 1; Additional file 6: Table S7), both resulting from influences by the more active biosyntheses of GAs and steroids in FCG root than in MCG’s.

Almost all studies on the transcriptome of *P. ginseng* have referred to the biosynthesis of ginsenosides. Gao et al. [39] identified 248 genes related to ginsenosides biosynthesis and 257 defense-responsive genes in FCG upon infection by *Cylindrocarpon destructans*, and found that 39 of the former showed a relationship with 29 of the latter. In adventitious roots of FCG treated with methyl jasmonate, Cao et al. [40] identified not only 749 ginsenosides biosynthesis genes, but also 12 pleiotropic drug resistance genes that were related to ginsenosides transportation. Liu et al. [41] further reported that *P. notoginseng* down-regulated biosynthesis of ginsenosides in response to arsenic stress. Lee and Mudge [42] reported that water deficit could contribute to an increasing content of ginsenosides in American ginseng. These findings revealed that ginsenosides biosynthesis could be affected by both biotic and abiotic stresses, whereby it can explain why FCG and MCG exhibited a different level of ginsenosides in their roots. Following this line of arguments, we suggest that an optimal growth condition favoring FCG cultivation might produce more dammarenediol II and PPD-type ginsenosides, whilst a harsh growth environment may force MCG under the natural condition to transform more PPD-type into PPT-type ginsenosides (Fig. 4d). Accordingly, we conjectured that it is PPD-type that is sensitive to stresses, and PPT-type ginsenosides might respond lately than PPD-type ginsenosides did when the environment stresses were further accentuated. This conjecture could explain a phenomenon [43] that intermittent chilling (25 °C for 12 h and 5 °C for 12 h) increased biosynthesis of PPD-type ginsenosides whereas continuous chilling (5 °C for 24 h) up-regulated both of PPD- and PPT-type ginsenosides in that PPD-type ginsenosides might be produced initially for responding to chilling stress, and continuous chilling would lead to a conversion of redundant PPD-type into PPT-type ginsenosides.

## Conclusions

Overall, this study firstly provided an overview of terpenoids metabolism in roots of FCG and MCG. Hereinto, diterpenoids and triterpenoids biosyntheses genes were significantly higher-expressed in FCG root, and conversely, carotenoid biosyntheses were more active in MCG root, and additionally, biosyntheses of terpenoid backbone, monoterpenoid, sesquiterpenoid and terpenoid-quinones were not obviously inclined in either types. To be specific, it was detected that there were higher expressions of GAs and steroids biosyntheses genes in FCG root that might be responsible for its quick vegetable growth, and higher levels of ABA and germacrenes as well as protopanaxatriol-type ginsenosides were conductive to enhance stress-resistance in MCG root. Our findings could greatly contribute to elucidate the underlying mechanisms for the different morphological appearances and phytochemical compositions between roots of FCG and MCG, and moreover, offered referable points of view for understanding terpenoids biosyntheses in other cultivated and wild species.

## Methods

### Plant materials

Five-year-old field-cultivated ginseng (FCG) and mountain-cultivated ginseng (MCG) (*P. ginseng* cv. Ermaya) were collected in late September, 2016, from a field plot (125°2′32.14″E, 41°2′40.92″N) of Kuandian county, Liaoning province, China, and a neighboring mountain plot (125°37′32.92″E, 41°2′39.85″N) of Huanren county, Liaoning province, China. Average annual temperature and rainfall of the field plot were 7.13 °C and 1051.1 mm, and those of mountain plot were 6.93 °C and 814.5 mm (For details, see Additional file 7: Table S1).

Lengths and diameters of stem, flower stalk, petiole, tap and lateral roots as well as lengths and widths of leaves from each individual ginseng plant were recorded with vernier calipers upon collection. After cleaning with distilled water on the spot, ginseng roots were immediately frozen in liquid nitrogen.

### Isolation, library construction and sequencing of RNA

Total RNAs from roots of FCG and MCG were isolated using Trizol reagent (Thermo Fisher Scientific Inc., Waltham, USA) according to the manufacturer’s protocol. Briefly, an aqueous phase was used for RNA precipitation with an equal volume of isopropanol. The RNA pellet was washed once with 1 mL 75% ethanol, then re-dissolved in an appropriate volume of RNase-free water. For each of the two samples, at least three individual ginseng roots were collected for RNA isolation. To avoid interference by proteins and polysaccharides, RNA concentration and quality were evaluated using a ND-2000 spectrophotometer (Thermo Fisher Scientific Inc., Waltham, USA) and a 2100 Bioanalyzer (Agilent Technologies Inc., Santa Clara, USA), respectively. According to the TruSeq RNA sample preparation guide (Illumina Inc., San Diego, USA), all these two samples should display a ratio of absorbance at 260 nm to that at 280 nm (*R*_260/280_) at 1.8–2.0, and a RNA integrity number being lager than or equal to 8.0.

Five μg of total RNA for each of the two ginseng root samples was used for construction of libraries using the TruSeq RNA sample preparation Kit. Briefly, poly (A)^+^ mRNA was isolated using magnetic Oligo-dT beads (Thermo Fisher Scientific Inc., Waltham, USA) and fragmented randomly into 200 bp in fragmentation buffer. The constructed DNA template was enriched by PCR amplification of 15 cycles. Amplicons were collected and purified by the Certified Low Range Ultra Agarose (Bio-Rad Inc., Hercules, USA) gel electrophoresis. High-throughput sequencing was performed on an Illumina Hiseq4000 sequencer (Illumina Inc., San Diego, USA) using a Truseq SBS Kit v3-HS 200 cycles Kits (Illumina Inc., San Diego, USA). The Illumina RNA-sequencing raw reads are available from the NCBI Sequence Read Archive database (http://www.ncbi.nlm.nih.gov/sra) under the accession number of PRJNA419783.

### Function annotation

After obtaining data of sequencing, raw reads were cleaned by removing adapter and low quality sequences using SeqPrep and Sickle 1.2 software, yielding 79,251 million clean reads that reached a total length of 10.83 Gbp (occupying 95.08% of the raw reads). Then, the obtained clean reads were referred onto the genome database of a ginseng cultivar Chunpoong (Ginseng Genome Database, http://ginsengdb.snu.ac.kr) [44] with the aid of Hisat2 software [45]. Based on this genome, we calculated the sequencing depth of the present transcriptome data was 6G (about 190X). Gene function annotations to databases including NR, Swissprot, Pfam, GO, COG, and KEGG were carried out using a combination of softwares such as Diamond v0.8.37.99, Hmmer 3.1b2, Blast2GO 2.5.0 and Kobas 2.1.1. Transcription factors (TFs) were annotated and classified using Hmmscan based on the PlantTFDB database.

### Analysis of differentially expressed genes

Expression of the annotated genes was profiled by the values of fragments per kilobase of transcript per million mapped reads (FPKM), and differentially expressed genes (DEGs) were identified by comparing their FPKM values through the DEGseq 30.0 (i.e., if *P* < 0.05 and the false discovery rate < 0.05, then the result is considered statistically significant.). Subsequently, enrichment analysis of KEGG pathways was performed based on these DEGs using KOBAS software.

### Validation by qRT-PCR

The genes of interest were subjected to quantitative real-time PCR (qRT- PCR) analysis. Among the DEGs, nine genes were randomly chosen for their drastically differential expressions or for their involvement in biosynthesis of terpenoids. The *IF3G1* (KU215663.1) gene was used as the internal reference according to a previous study [46]. Primers were listed in Additional file 8: Table S2. Approximately 1.0 μg of total RNA from each of ginseng root samples was reverse-transcribed using a Rayscript cDNA Synthesis Kit (GENEray Biotech Co., Ltd., Shanghai, China). Subsequently, amplification was performed through a SYBR Green I method using 0.5 μl (10 μM) of specific primers, 5 μl cDNA (750 ng/μL), and 4.5 μl PCR Enzyme Mix from a Power qPCR PreMix Kit (GENEray Biotech Co., Ltd., Beijing, China). Cycling parameters were 95 °C for 5 min followed by 40 cycles at 95 °C for 10 s, 60 °C for 34 s and 90 °C for 15 s. Three independent biological and technological replicates were performed. Amplification specificity was assessed by dissociation curve analysis, and relative gene expression was analyzed using the 2^-ΔΔct^ method [47].

### Determination of key terpenoids

#### Measurements of ABA and GA by spectrophotometry

Extraction of abscisic acid (ABA) was conducted as follows: 1.000 g fresh ginseng root was ground in liquid nitrogen with a mortar, then sonicated (KQ5200E sonicator, Kunshan Instrument Co., Ltd., Kunshan, China) for 15 min in 3 mL cold extracting solvent [acetone: water: acetic acid, 80:19:1 (v:v:v)]. Subsequently, the mixture was centrifuged (Sigma, St. Louis, USA) at 4000 rpm and 4 °C for 10 min. The supernatant was collected and the residue was re-extracted twice more. The three supernatants were combined and diluted to 10 mL with the extracting solvent for analysis. Determination of ABA was carried out with enzyme-linked immunosorbent assay (ELISA Kit, Coolaber Co., Ltd., Beijing, China) by following the manufacturer’s instructions.

Extraction of gibberellins (GAs) were conducted as follows: 0.500 g fresh ginseng root was sonicated for 5 min in 5 mL cold water, then centrifuged for 10 min at 5000 rpm and 4 °C. The supernatant was collected and the residue was re-extracted twice more. Afterwards, three supernatants were combined and diluted to 15 mL with water. After adjusting pH to 2.8 with 15% acetic acid, the aqueous solution was partitioned against 15 mL ethyl ether, and the upper organic layer was collected and completely evaporated to dryness at room temperature, which was finally resuspended in 1 mL 10% aqueous methanol for analysis. GAs were determined by an enzyme-linked immunosorbent assay (ELISA Kit, Coolaber Co., Ltd., Beijing, China).

#### Measurements of ginsenosides by HPLC

Ginsenosides were isolated and determined according to a previous report [48] with slight modifications. Fresh ginseng root of 1.000 g was pulverized and added into 10 mL 70% aqueous ethanol. The mixture was sonicated for 40 min in cold water, then filtered with a 0.22-μm filter (Keyilong Instrument Co., Ltd., Tianjin, China). The filtrates were collected and the residual was re-extracted twice more. Finally, three filtrates were combined and evaporated to dryness at 40 °C using a rotary evaporator (RE52-A, Yarong Instrument Co., Ltd., Shanghai, China). Dried extract was dissolved in 5 mL methanol and filtered through a 0.22-μm syringe filter prior to HPLC analysis. For HPLC analysis, a Shimadzu HPLC system (Shimadzu Corp., Kyoto, Japan) equipped with a SPDM20A ultraviolet detector, a Diamonsil 5 μm C_18_ (II) column (250 × 4.6 mm column, Dikma Tech. Inc., Beijing, China), and a SIL-20 AC TH autosampler controlled by an analytical software (LC Solution-Release 1.23SP1) were applied. Determination of ginsenosides was conducted under the following conditions: the mobile phase was 0.1% (*v*/v) phosphoric acid in water (A) and acetonitrile (B) (A: B = 80: 20) and gradient elution programme was 0–14 min, 21% B; 14–30 min, 21–30% B; 30–60 min, 30–32% B; 60–75 min, 32–33% B; 75–100 min, 33–35% B; 100–110 min, 35–37% B; 110–120 min, 37–60% B; 120–130 min, 60–70% B; 130–140 min, 70–80% B; 140–150 min, 80–100% B. Monitoring wavelength was set at 203 nm, flow rate at 1.0 mL/min, and column thermostat at 25 °C. Ginsenosides standards were purchased from National Institutes for Food and Drug Control (Beijing, China), and stored at − 20 °C until analysis. A profile of standards (protopanaxadiol types: Rg_1_ 1 mg/ml, Re 0.75 mg/ml, Rf 0.3 mg/ml, F_1_ 1.2 mg/ml, and Rg_2_ 0.2 mg/ml; protopanaxatriol types: Rb_1_ 0.3 mg/ml, Rc 0.25 mg/ml, Rb_2_ 0.25 mg/ml, Rb_3_ 0.2 mg/ml, Rd. 0.3 mg/ml, and Rh_2_ 0.25 mg/ml) in methanol was shown in Additional file 9: Figure S1. Relative coefficients of calibration curves for all standards were above 0.998.

#### Measurements of several other terpenoids by GC-MS

To reduce the loss of violated terpenoids, supercritical CO_2_ extraction (SFE) was performed. About 3 g of lyophilized samples was charged in a 10-cm^3^ vessel at each run. The flow rate of CO_2_ was 1.5 L/h, and 30 ml ethanol was used as entrainer. After preliminary tests, the extracting time was determined as 120 min, and the temperature and pressure in the vessel were set at 45 °C and 400 bar.

Target terpenoids were separated and quantified using a Shimadzu GC-MS-QP2010 Plus (Shimadzu, Kyoto, Japan) equipped with a Rtx-5MS column (30 m × 0.25 mm, 0.25 μm film thickness) (Shimadzu, Kyoto, Japan). GC conditions used were set as follows: programmed heating from 40 to 320 °C at 5 °C /min followed by 10 min under isothermal conditions. The injection port and detector were set at 300 °C. Helium was used as carrier gas in a constant flow at 1.0 ml/min. A 10-μl of SFE sample was injected in the split mode (1:10). MS conditions were as follows: ionization energy, 70 eV; ion source temperature, 220 °C; mass range, 40–750 m/z. The NIST Special Database 14 was used as references of mass spectra.

#### Statistical analysis

Experiments on all determinations were carried out in triplicate, and results were presented as means ± stand deviation (SD). The statistical analysis was done by the SPSS 19.0 software, and *P* value < 0.05 was considered to be statistically significant.

## Additional files

Additional file 1 **Table S3.** Quality control of sequencing. (DOCX 14 kb)

Additional file 2 **Table S4.** Distribution of clean reads mapped to reference genome. (DOCX 13 kb)

Additional file 3 **Table S5.** Tentative identification of terpenoids by GC-MS. (DOCX 17 kb)

Additional file 4 **Figure S2.** GC-MS total ion chromatography of MCG and FCG roots. (TIF 31295 kb)

Additional file 5 **Table S6.** Distribution of annotated transcription factors in TF family. (XLS 31 kb)

Additional file 6 **Table S7.** Morphological characterization of field- and mountain-cultivated ginsengs. (DOCX 16 kb)

Additional file 7 **Table S1.** Monthly average temperature and rainfall of Kuandian county. (DOCX 15 kb)

Additional file 8 **Table S2.** Primers of nine key genes in terpenoids biosyntheses for qPCR. (DOCX 14 kb)

Additional file 9 **Figure S1.** HPLC profiles of 11 ginsenosides standards. (JPG 91 kb)

## Abbreviations

(−)-α-TS: (−)-alpha-terpineol synthase
(+)-ND: (+)-neomenthol dehydrogenase
AAO3: Abscisic-aldehyde oxidase
ABA: Abscisic acid
ABA2: Xanthoxin dehydrogenase
ABAH: (+)-abscisic acid 8′-hydroxylase
ACT: Acetyl-CoA C-acetyltransferase
CAS: Cycloartenol synthase
CCD7: 9-cis-beta-carotene 9′,10′-cleaving dioxygenase
CCD8: Carlactone synthase
CCS1: Capsanthin/capsorubin synthase
CPI1: Cycloeucalenol cycloisomerase
crtB: 15-cis-phytoene/all-trans-phytoene synthase
crtISO: Prolycopene isomerase
CYP51: Sterol 14-demethylase
CYP710A: Steroid 22-desaturase
CYP71D55: Premnaspirodiene oxygenase
CYP724B1: Cytochrome P450 family 724 subfamily B polypeptide 1
CYP734A1: PHYB activation tagged suppressor 1
CYP85A2: Cytochrome P450 family 85 subfamily A2
CYP90A1: Cytochrome P450 family 90 subfamily A polypeptide 1
CYP90B1: Steroid 22-alpha-hydroxylase
CYP90D1: Cytochrome P450 family 90 subfamily D1
DEGs: Differentially expressed genes
DET2: Steroid 5-alpha-reductase
DHCR24: Delta24-sterol reductase
DHCR7: 7-dehydrocholesterol reductase
DMAPP: Dimethylallyl pyrophosphate
DS: Dammarenediol-II synthase
DWARF27: Beta-carotene isomerase
DXR: 1-deoxy-D-xylulose-5-phosphate reductoisomerase
DXS: 1-deoxy-D-xylulose-5-phosphate synthase
EBP: Cholestenol Delta-isomerase
*ent*-KS: *ent*-kaurene synthase
ERG3: Delta 7-sterol-5-desaturase
FCG: Field-cultivated ginseng
FDFT1: Farnesyl-diphosphate farnesyltransferase
FDPS: Farnesyl diphosphate synthase
FDR: False discovery rate
FK: Delta14-sterol reductase
FLDH: NAD^+^ −dependent farnesol dehydrogenase
FNTA/B: Alpha/beta-protein farnesyltransferase
FOLK: Farnesol kinase
FPKM: Fragments per kilobase of transcript per million mapped reads
FPP: Farnesyl diphosphate
G3P: Glyceraldehyde 3-phosphate
GA20ox: Gibberellin 20-oxidase
GA2ox: Gibberellin 2-oxidase
GA3ox1: Gibberellin 3-beta-dioxygenase
GAs: Gibberellins
GERD: Germacrene synthase
GGPP: Geranylgeranyl pyrophosphate
GGPS: Geranylgeranyl diphosphate synthase
GPP: Geranyl diphosphate
HMGCR: Hydroxymethylglutaryl-CoA reductase
HMGS: Hydroxymethylglutaryl-CoA synthase
HPT: Homogentisate phytyltransferase
HST: homogentisate solanesyltransferase
IDI: Isopentenyl-diphosphate delta-isomerase
ispD: 2-C-methyl-D-erythritol 4-phosphate cytidylyltransferase
ispG: (*E*)-4-hydroxy-3-methylbut-2-enyl-diphosphate synthase
ispH: 4-hydroxy-3-methylbut-2-en-1-yl diphosphate reductase
KAO: Ent-kaurenoic acid hydroxylase
lcyB: Lycopene beta-cyclase
LUT5: Beta-ring hydroxylase
MCG: Mountain-cultivated ginseng
menA: 1,4-dihydroxy-2-naphthoate octaprenyltransferase
MEP/DOXP: 2-*C*-methyl-D-erythritol 4-phosphate/1-deoxy-D-xylulose 5-phosphae
MVA: Mevalonic acid
mvaD: Diphosphomevalonate decarboxylase
mvaK1: Mevalonate kinase
mvaK2: Phosphomevalonate kinase
NCED: 9-cis-epoxycarotenoid dioxygenase
PCYOX1: Prenylcysteine oxidase
PD: 15-cis-phytoene desaturase
PDS: Protopanaxadiol synthase
PPD: Protopanaxadiol
PPT: Protopanaxatriol
prephytoene-PP: Prephytoene diphosphate
PTS: Protopanaxatriol synthase
qRT- PCR: Quantitative real-time PCR
SMO1: 4,4-dimethyl-9beta,19-cyclopropylsterol-4alpha-methyl oxidase
SMO2: 4-alpha-methyl-delta7-sterol-4alpha-methyl oxidase
SMT1: Sterol 24-C-methyltransferase
SMT2: 24-methylenesterol C-methyltransferase
SQLE: Squalene monooxygenase
TOMT: Tocopherol O-methyltransferase
TPS1: Valencene/7-epi-alpha-selinene synthase
ubiE: Demethylmenaquinone methyltransferase
VDE: Violaxanthin de-epoxidase
VTE1: Tocopherol cyclase
VTE3: 2-methyl-6-phytyl-1,4-hydroquinone methyltransferase
wrbA: NAD(P)H dehydrogenase
ZDS: Zeta-carotene desaturase
ZEP: Zeaxanthin epoxidase
*β*-AS: Beta-amyrin synthase
