# Supplementary material for: Comparative transcriptome analyses on terpenoids metabolism in field- and mountain-cultivated ginseng roots
Source: BMC Plant Biol. 2019 Feb 19;19:82. doi: 10.1186/s12870-019-1682-5 (PMC6381674; doi:10.1186/s12870-019-1682-5)
Supplement: Supplementary file 1 — Table S3. Quality control of sequencing. (DOCX 14 kb) [file 12870_2019_1682_MOESM1_ESM.docx]

Additional file 4: Table S3 Quality control of sequencing

| Sample | Clean reads | Clean bases | Error rate (%) | Q20 (%) | Q30 (%) | GC content (%) |
| --- | --- | --- | --- | --- | --- | --- |
| FCG | 35239768 | 5167440891 | 0.00103 | 98.34 | 95.29 | 44.53 |
| MCG | 44102034 | 6469421038 | 0.00101 | 98.40 | 95.46 | 43.26 |
