# Supplementary material for: Comparative transcriptome analyses on terpenoids metabolism in field- and mountain-cultivated ginseng roots
Source: BMC Plant Biol. 2019 Feb 19;19:82. doi: 10.1186/s12870-019-1682-5 (PMC6381674; doi:10.1186/s12870-019-1682-5)
Supplement: Supplementary file 2 — Table S4. Distribution of clean reads mapped to reference genome. (DOCX 13 kb) [file 12870_2019_1682_MOESM2_ESM.docx]

Additional file 5: Table S4 Distribution of clean reads mapped to reference genome

| Sample | MCG | FCG |
| --- | --- | --- |
| Introns | 2643510(4.6%) | 1945065(4.5%) |
| 3'UTR | 823761(1.43%) | 692695(1.6%) |
| CDS | 47739359(83.16%) | 35892735(83.11%) |
| 5'UTR | 508795 (0.89%) | 469517(1.09%) |
| Intergenic | 5694048 (9.92%) | 4186879(9.69%) |
