# Supplementary material for: Comparative transcriptome analyses on terpenoids metabolism in field- and mountain-cultivated ginseng roots
Source: BMC Plant Biol. 2019 Feb 19;19:82. doi: 10.1186/s12870-019-1682-5 (PMC6381674; doi:10.1186/s12870-019-1682-5)
Supplement: Supplementary file 3 — Table S5. Tentative identification of terpenoids by GC-MS. (DOCX 17 kb) [file 12870_2019_1682_MOESM3_ESM.docx]

Additional file 6: Table S5. Tentative identification of terpenoids by GC-MS

| RT (FCG) | RT (MCG) | Area (FCG) | Area (MCG) | CAS | Compounds | Name | MW | Ion peaks |
| --- | --- | --- | --- | --- | --- | --- | --- | --- |
|  | 20.671 |  | 255378 | 25246-27-9 | C_15_H_24_ | alloaromadendrene | 204 | 161/133/119/105/91 |
|  | 20.879 |  | 170850 | 515-13-9 | C_15_H_24_ | beta-elemene | 204 | 161/147/133/93/81 |
| 21.896 | 21.891 | 292670 | 718309 | 17334-55-3 | C_15_H_24_ | calarene | 204 | 189/161/147/133/105 |
|  | 21.992 |  | 289903 | 56633-28-4 | C_15_H_24_ | alpha-panasinsene | 204 | 161/122/107/91/81 |
|  | 23.026 |  | 247774 | 56684-96-9 | C_15_H_24_ | beta-neoclovene | 204 | 189/161/147/133/105 |
|  | 23.167 |  | 153421 | 23986-74-5 | C_15_H_24_ | germacrene D | 204 | 161/119/105/91 |
|  | 23.353 |  | 158051 | 489-39-4 | C_15_H_24_ | aromandendrene | 204 | 161/133/105/91/79 |
|  | 23.436 |  | 138457 | 21747-46-6 | C_15_H_24_ | ledene | 204 | 147/133/119/107/93 |
| 23.555 | 23.539 | 505751 | 853575 | 29873-99-2 | C_15_H_24_ | gamma-elemene | 204 | 133/121/107/93/79 |
| 29.187 |  | 460033 |  | 25061-77-2 | C_14_H_12_O_2_ | 9,10-phenanthrenediol | 212 | 212/194/181/165/152 |
| 31.846 |  | 245272 |  | 61142-32-3 | C_10_H_18_ | 1,3-dimethyl-2-(1-methylethyl)-cyclopentene | 138 | 123/95/81 |
| 32.320 | 32.363 | 338937 | 433366 | 99-82-1 | C_10_H_20_ | p-menthane | 140 | 138/123/95/81 |
| 37.816 | 37.831 | 924063 | 446038 | 72800-72-7 | C_17_H_24_O_2_ | panaxydol | 260 | 133/121/91/77/55 |
| 45.81 | 45.808 | 537421 | 326599 | 67-96-9 | C_28_H_46_O | dihydrotachysterol | 398 | 255/147/133/121/93 |
|  | 48.442 |  | 156180 | 1686-54-0 | C_21_H_32_O_2_ | 1-phenanthrenecarboxylic acid | 316 | 180/121/105/91 |
| 48.596 | 48.594 | 242461 | 165463 | 83-48-7 | C_29_H_48_O | stigmasta-5,22-dien-3-ol | 412 | 255/159/83/69/55 |
| 49.37 | 49.368 | 792614 | 738136 | 915-05-9 | C_31_H_52_O_2_ | stigmast-5-en-3-ol, acetate | 456 | 255/159/147/95/81 |
|  | 49.464 |  | 185001 | 23445-02-5 | C_15_H_26_O | cubebol | 222 | 207/161/119/105 |
| 50.269 | 50.269 | 1874203 | 1665787 | 4651-48-3 | C_31_H_50_O_2_ | stigmasta-5,22-dien-3-ol, acetate | 454 | 395/255/145/81/69 |
| 50.614 | 50.613 | 1145268 | 1034088 | 7144-08-3 | C_28_H_45_ClO_2_ | cholesterol chloroformate | 448 | 147/105/91/81 |
|  | 51.063 |  | 5954181 | 79897-80-6 | C_29_H_48_ | stigmasta-3,5-diene | 396 | 213/147/133/105/81 |
| 51.305 | 51.31 | 278698 | 202220 | 473-98-3 | C_30_H_50_O_2_ | lup-20(29)-ene-3,28-diol | 442 | 203/189/135/107/95 |
| 51.689 | 51.682 | 408612 | 209862 | 59-02-9 | C_29_H_50_O_2_ | tocopherol | 430 | 165/121 |
| 53.132 | 53.12 | 850243 | 531738 | 57-87-4 | C_28_H_44_O | ergosterol | 396 | 253/143/69/55 |
| 53.276 | 53.273 | 406005 | 160778 | 1449-09-8 | C_31_H_52_O | 24-methylenecycloartano | 440 | 203/175/135/95/81 |
| 53.883 | 53.879 | 2336748 | 1582292 | 83-47-6 | C_29_H_50_O | gamma-sitosterol | 414 | 213/159/145/107/95 |
| 54.048 | 54.048 | 470769 | 222714 | 1686-63-1 | C_20_H_30_O | isopimaral | 286 | 187/131/119/105/91 |
| 54.297 |  | 362814 |  | 1617-68-1 | C_32_H_52_O_2_ | lupeol acetate | 468 | 218/203/189/135/95 |
| 54.441 | 54.481 | 833734 | 261274 | 638-95-9 | C_30_H_50_O | alpha-amyrin | 426 | 218/203/189/135/122 |
| 54.938 | 54.856 | 456709 | 107646 | 2034-72-2 | C_29_H_46_O | stigmasta-3,5-dien-7-one | 410 | 187/174/161 |
| 55.388 | 55.347 | 1216091 | 628026 | 51014-22-3 | C_28_H_46_O | 4-campestene-3-one | 398 | 229/124/107/93 |
| 59.327 | 59.172 | 175019 | 268790 | 29790-47-4 | C_50_H_70_O_2_ | 1,4-naphthoquinone | 702 | 253/239/225/211 |
| 59.486 | 59.483 | 313216 | 266352 | 1259-10-5 | C_32_H_52_O_2_ | cycloartenol acetate | 468 | 147/135/109/95/69 |

RT: rention time (min). MW: molecular weight.
