# Supplementary material for: Comparative transcriptome analyses on terpenoids metabolism in field- and mountain-cultivated ginseng roots
Source: BMC Plant Biol. 2019 Feb 19;19:82. doi: 10.1186/s12870-019-1682-5 (PMC6381674; doi:10.1186/s12870-019-1682-5)
Supplement: Supplementary file 6 — Table S7. Morphological characterization of field- and mountain-cultivated ginsengs. (DOCX 16 kb) [file 12870_2019_1682_MOESM6_ESM.docx]

Additional file 8: Table S7. Morphological characterization of field- and mountain-cultivated ginsengs (FCG and MCG) (mm)

|  | | Overground parts | | | | Underground parts | |
| --- | --- | --- | --- | --- | --- | --- | --- |
|  |  | Leaf ^a^ | Stem | Flower stalk | Petiole ^b^ | Tap root | Lateral root |
| FCG | Length | 178.08±9.62 | 793.67±23.29 | 354.67±39.50 | 122.87±9.00 | 206.00±7.81 | 7.00±1.00 |
|  | Diameter | 74.57±10.95^c^ | 9.88±1.72 | 4.13±0.62 | 7.06±1.54 | 25.99±0.93 | 12.90±2.65 |
| MCG | Length | 123.60±6.56 | 700.67±11.02 | 253.33±15.28 | 83.07±1.67 | 263.33±37.86 | 12.33±0.58 |
|  | Diameter | 54.67±3.17 ^c^ | 6.50±0.77 | 2.13±0.49 | 3.76±1.08 | 16.32±1.78 | 5.77±1.33 |

The values were presented as means ± SD. Except leaf and petiole, other parts were calculated by means of three replicates. All data between the two types of ginseng roots exhibited a significant difference (*P* < 0.05).

^a)^ The value was calculated by means of 45 main leaves from fifteen palmately compound leaves.

^b)^ The value was calculated by means of 25 petioles from fifteen palmately compound leaves.

^c)^ The values were the calculated by width of leaves.
