# Supplementary material for: Comparative transcriptome analyses on terpenoids metabolism in field- and mountain-cultivated ginseng roots
Source: BMC Plant Biol. 2019 Feb 19;19:82. doi: 10.1186/s12870-019-1682-5 (PMC6381674; doi:10.1186/s12870-019-1682-5)
Supplement: Supplementary file 7 — Table S1. Monthly average temperature and rainfall of Kuandian county. (DOCX 15 kb) [file 12870_2019_1682_MOESM7_ESM.docx]

Additional file 1: Table S1 Monthly average temperatures, rainfalls and daylengths of the two sampling plots*

| Month | average temperature (°C) | | average  rainfall (mm) | | average daylength (h) |
| --- | --- | --- | --- | --- | --- |
|  | FCG | MCG | FCG | MCG | FCG and MCG |
| Jan. | -11.5 | -12.4 | 10.4 | 6.3 | 9.55 |
| Feb. | -7.4 | -8.2 | 12.5 | 7.3 | 10.65 |
| Mar. | 0.2 | 0.2 | 19.0 | 12.8 | 11.90 |
| Apr. | 8.3 | 8.8 | 50.7 | 40.6 | 13.30 |
| May. | 14.6 | 15.2 | 74.6 | 62.5 | 14.47 |
| Jun. | 19.4 | 19.9 | 129.7 | 111.5 | 15.10 |
| Jul. | 22.5 | 23.0 | 284.7 | 210.0 | 14.83 |
| Aug. | 22.2 | 22.1 | 274.8 | 210.0 | 13.83 |
| Sep. | 16.1 | 15.5 | 92.6 | 76.2 | 12.48 |
| Oct. | 8.8 | 8.3 | 52.2 | 44.4 | 11.15 |
| Nov. | 0.2 | -0.5 | 34.4 | 24.0 | 9.90 |
| Dec. | -7.8 | -8.7 | 15.5 | 8.9 | 9.23 |

* The data was obtained from China meteorological administration
