# Supplementary material for: Comparative transcriptome analyses on terpenoids metabolism in field- and mountain-cultivated ginseng roots
Source: BMC Plant Biol. 2019 Feb 19;19:82. doi: 10.1186/s12870-019-1682-5 (PMC6381674; doi:10.1186/s12870-019-1682-5)
Supplement: Supplementary file 8 — Table S2. Primers of nine key genes in terpenoids biosyntheses for qPCR. (DOCX 14 kb) [file 12870_2019_1682_MOESM8_ESM.docx]

Additional file 2: Table S2 Primers of nine key genes in terpenoids biosyntheses for qPCR

| Genes |  | Primers |
| --- | --- | --- |
| *ABA2* | F | CCAGAAGACCCTGAGTGAGT |
|  | R | TAGCCAGTGATGAAGCAAAG |
| *GA20OX* | F | ATTTCAGCGCATTTGTTGT |
|  | R | CACCTTATCCTCCTTTGGG |
| *HMGCR* | F | TTTTACGGATGTGGTGGAG |
|  | R | TCGTCTTCGTCTATGATGGTT |
| *SQLE* | F | TAAAAGGGGTGCGATACAA |
|  | R | TCAAACCAACAAAACACGAC |
| *VTE3* | F | TTTCTCTTGCGTTTCGTTC |
|  | R | ATGTTTTCTTCTGGCTTTTCA |
| *(+)-ND* | F | AATAACAAGGGAAACACCACA |
|  | R | TACAAGATGAGAAAGCAACACA |
| *FDPS* | F | TTGAATACGGAGGAATGAGAG |
|  | R | TGGAAGTGGTAAAGGGGTT |
| *GA3OX1* | F | TAGGAGCAGGAGTTGGTAGG |
|  | R | TGTATGTGGCATGAAGGGT |
| *GA2ox* | F | GATTTGTGGGTCAGTATGTTCTC |
|  | R | TTTAGTGAGCTTTTGATGGATG |
| *IF3G1* | F | CCAACCTCGGATACAACAA |
|  | R | GTTCTCCTCATTCCTGCGT |
