# Supplementary figures and images for: Comparative transcriptome analyses on terpenoids metabolism in field- and mountain-cultivated ginseng roots
Source: BMC Plant Biol. 2019 Feb 19;19:82. doi: 10.1186/s12870-019-1682-5 (PMC6381674; doi:10.1186/s12870-019-1682-5)

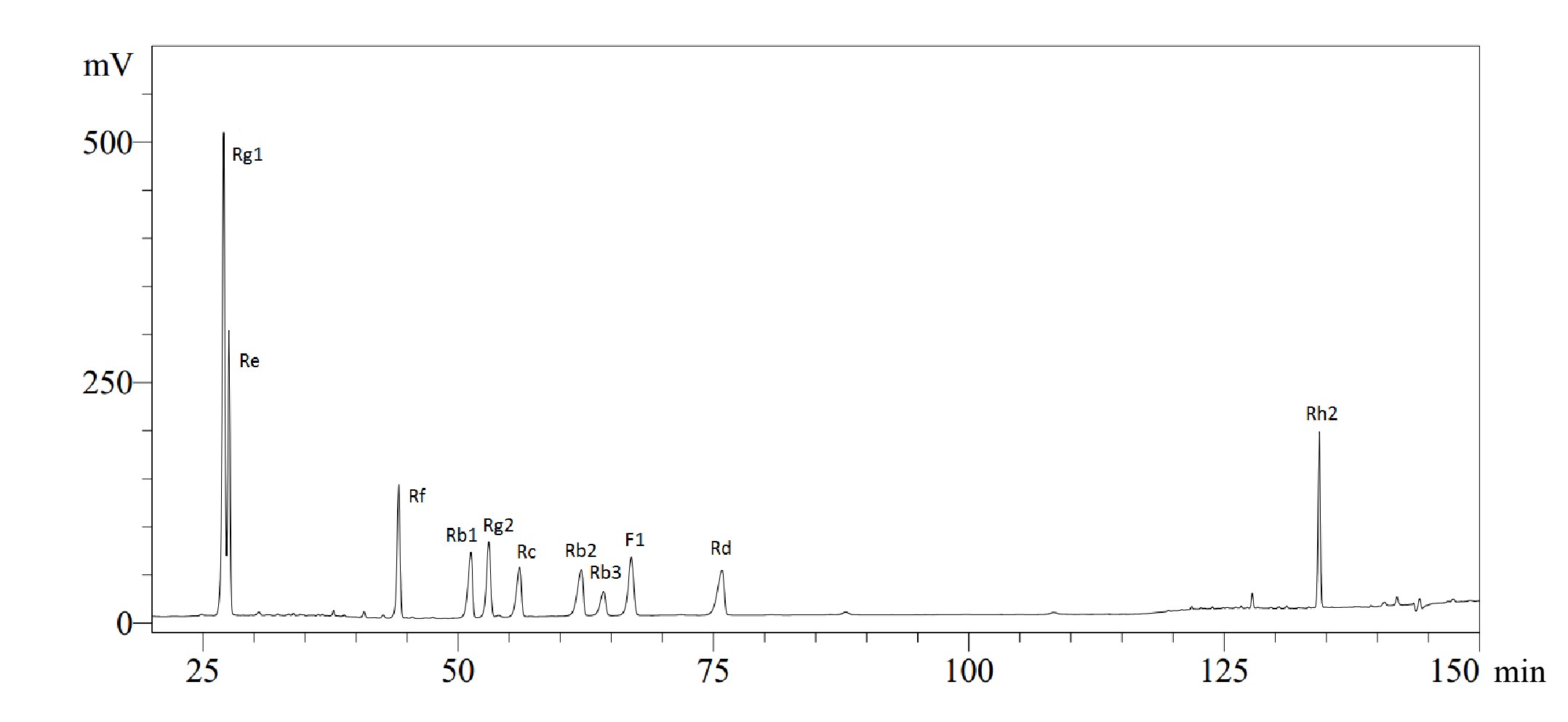

Supplement: Supplementary file 9 — Figure S1. HPLC profiles of 11 ginsenosides standards. (JPG 91 kb) [file 12870_2019_1682_MOESM9_ESM.jpg]
